# Assessment and Management of Ischaemic Heart Disease in Non-Cardiac Surgery

**DOI:** 10.17925/HI.2023.17.2.19

**Published:** 2023-12-01

**Authors:** Holly Morgan, Saad M Ezad, Haseeb Rahman, Kalpa De Silva, Judith S L Partridge, Divaka Perera

**Affiliations:** 1. British Heart Foundation Centre of Research Excellence at the School of Cardiovascular and Metabolic Medicine and Sciences, King's College, London, UK; 2. Guy’s and St Thomas’ NHS Foundation Trust, London, UK

**Keywords:** Coronary syndrome, ischaemic heart disease, non-cardiac surgery, perioperative, myocardial injury, coronary revascularization

## Abstract

In the setting of non-cardiac surgery, cardiac complications contribute to over a third of perioperative deaths. With over 230 million major surgeries performed annually, and an increasing prevalence of cardiovascular risk factors and ischaemic heart disease, the incidence of perioperative myocardial infarction is also rising. The recent European Society of Cardiology guidelines on cardiovascular risk in noncardiac surgery elevated practices aiming to identify those at most risk, including biomarker monitoring and stress testing. However the current evidence base on if, and how, the risk of cardiac events can be modified is lacking. This review focuses on patient, surgical and cardiac risk assessment, as well as exploring the data on perioperative revascularization and other risk-reduction strategies.

Cardiac complications constitute a common cause of morbidity and mortality in the perioperative period, contributing to over a third of perioperative deaths.^1^ More than 230 million major surgeries are performed annually worldwide, with older patients representing an increasing proportion of the surgical population; a group with a significant burden of cardiovascular risk factors and a higher incidence of perioperative myocardial infarction (MI).^2^ These patients may develop major cardiovascular events precipitated by non-cardiac surgery (NCS) and in the longer term, as a manifestation of the natural history of their cardiovascular disease. Unrecognized or inadequately managed ischaemic heart disease (IHD) can lead to adverse perioperative outcomes including myocardial injury or infarction, heart failure, arrhythmia, prolonged hospital stay and increased mortality rates.^3,4^ Therefore, an integrated approach to assess, optimize, and manage perioperative IHD is essential to minimize risks and improve patient outcomes. The management of perioperative IHD presents several challenges. There is a need to accurately identify patients at high risk of complications, optimize modifiable risk factors and tailor management strategies accordingly. Additionally, balancing the risks and benefits of surgical procedures and considering the timing of surgery in relation to cardiac co-morbidity is crucial.

This review aims to provide an up-to-date understanding of the optimal strategies for managing perioperative IHD, taking into account risk assessment, preoperative optimization, pharmacological interventions, surgical considerations, postoperative care and emerging strategies.

## Myocardial injury after non-cardiac surgery

The leading concern for patients with known or suspected IHD undergoing NCS is MI, however the 4th Universal Definition of MI requires evidence of ischaemia (symptoms or electrocardiogram [ECG] changes) in addition to troponin elevation above the 99th upper reference limit centile.^5^ Trials in NCS have traditionally required ST changes or development of Q waves in addition to biomarker elevation. However, in the perioperative setting, ECG changes may be transient and are often not captured, whilst Q waves are known to be an inaccurate measure of adjudicating the significance of myocardial injury.

Diagnosis of myocardial injury after noncardiac surgery (MINS) is a more contemporary term relying upon a biomarker elevation post surgery, with the magnitude of injury correlating with mortality.^2^ MINS events are termed perioperative if they occur intraoperatively or within 30 days of surgery.^4^

**Figure 1:**
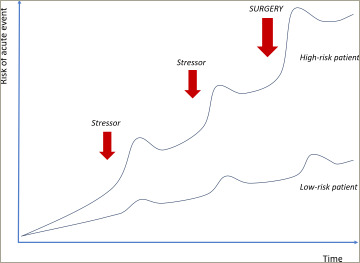
Risk of acute cardiac events when exposed to stressor

Perioperative MINS can occur due to type 1 and type 2 MI. Type 1 MI is caused by a stress-i nduced plaque rupture or erosion of an atherosclerotic plaque in combination with vascular inflammation and altered vasomotion.^4^ Type 2 MI is caused by a supply–demand mismatch of myocardial perfusion due to coronary artery stenoses that become flow-l imiting in the context of increased metabolic demand.^5^ Early mortality after MINS is up to 25%; the risk is proportionate to the degree of myocardial injury and troponin release.^6,7^ The detrimental cascade following perioperative MINS includes cardiogenic shock, leading to end-organ damage (including cerebral and renal hypoperfusion), each independently associated with greater morbidity and mortality (*Figure 1*).

**Table 1:** Comparison of three key guidelines on perioperative cardiac assessment^3,10,11^

	ESC 2022	CCS 2017	ACC/AHA 2014
**Preoperative assessment**			
**METS**	-	-	+
**Frailty assessments**	+	-	-
**Routine ECG**	+	+/-	+/-
**Routine biomarkers**	Trop, BNP	BNP	-
**Imaging**			
**Routine echo**	-	-	-
**CCTA**	+/-	-	-
**Stress testing**	In high/intermediate risk	-	In high risk/low METS
**Routine ICA**	-	-	-
**Interventions**			
**Revascularization**	Same indications as non-surgical setting	Consider if angina	Same indications as non-surgical setting
**DAPT period pre-NCS**	Minimum 1 month	Minimum 6 weeks	Minimum 1 month
**Postoperative monitoring**			
**Troponin surveillance**	^+^	^+^	-

*+ = recommended; - = not recommended*

*ACC = American College of Cardiology; AHA = American Heart Association; BNP = b-type natriuretic peptide; CCS = Canadian Cardiovascular Society; CCTA = coronary computed tomography angiography; DAPT = dual antiplatelet therapy; ECG = electrocardiogram; echo = echocardiogram; ESC = European Society of Cardiology; ICA = invasive coronary angiography; METS = metabolic equivalent of task; NCS = non-cardiac surgery; Trop = troponin.*

Histopathology specimens in cases of fatal perioperative MINS revealed that two-thirds of patients had significant left-main or three-vessel disease, implying type 2 MI, although prolonged states of myocardial oxygen supply–demand imbalance can also lead to plaque rupture.^8,9^ Whilst the mechanisms of MINS are well understood, accurate preoperative identification of patients at risk and management to modify this risk are less established.^4^

## Guidelines

Despite publications dating back to the 1970s, evidence for the prediction and prevention of perioperative major adverse cardiac events (MACE) remains sparse, and therefore variation exists in both clinical practice and guidelines (*Table 1*).^3,10,11^

## Preoperative risk assessment

The question to consider is: how does pre-surgical assessment differ from a general coronary assessment in a non-surgical patient? In both scenarios, it is key to assess and protect patients against the risk of potential events. Every time a patient is exposed to a stressor, for example an infection, a bleeding event, a drop in blood pressure, that causes a ‘jump’ in the potential of an acute event occurring.^8^ Given the risk of multiple stressors occurring at once, the biggest jump in event rate would be at the time of a surgical procedure (*Figure 1*).^8^

Many patients have stable coronary artery disease (CAD), but the key is identifying those who are at high risk of having an acute cardiac event when exposed to a surgical stressor, and how this risk can be modified.

**Figure 2:**
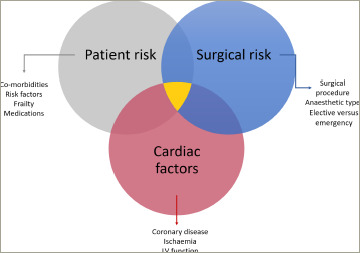
Three domains of perioperative cardiac risk

Risk factors for perioperative cardiac events can be considered as patient, operative or cardiac factors, only some of which may be modifiable (*Figure 2*).^3^

### Patient risk assessment

Patient-specific factors include sex, age, past medical history, nutritional status and symptom profile (*Figure 2*). Adequate control of all cardiovascular risk factors is vital; it has been identified in the non-surgical setting that cardiovascular prognosis and all-cause death significantly worsens as the number of risk factors not reaching therapeutic goal increases.^12^ Preoperative events will also impact risk, including recent cardiac events, as well as the acuity and severity of the surgical presentation. As a simple measure, documentation of observations, as well as a baseline ECG, should be standard, allowing for future comparison if required.^13^ Specialized cardiac risk assessment tools exist to guide surgeons and anaesthetists involved in preoperative care, including the Revised Cardiac Risk Index (RCRI) and the Acute Coronary Syndrome (ACS) National Surgical Quality Improvement Program (NSQIP) scores, which aims to predict risk specifically for MINS and/or cardiac arrest.^14,15^

#### Biomarkers

The recently updated European Society of Cardiology (ESC) guidelines advocate the preoperative measurement of biomarkers in patients with cardiac risk factors or symptoms; with a 1B recommendation for troponin.^3^ Preoperative troponin level also features in the revised cardiac index risk score, added after meta-analyses identified a correlation between perioperative troponin and incidence of MACE.^16,17^ Gualandro et al. found that both troponin I and T predict postoperative MACE, but troponin I had superior accuracy in patients undergoing vascular surgery.^18^ The Canadian Cardiovascular Society (CCS) 2017 guideline recommends a preoperative b-type natriuretic peptide (BNP) level in all patients undergoing surgery over the age of 65, or over 45 if they have cardiac risk factors.^9^ In a study of 10,402 patients aged over 45 years having inpatient NCS, preoperative BNP was strongly associated with MINS and mortality.^19^ Park et al. also reported that preoperative BNP level was more predictive of MACE risk than echocardiography.^20^ Of note, routine echocardiography is not recommended in the preoperative setting as it has not been found to improve perioperative outcomes.^21^ Despite this association, the implication for clinical practice remains uncertain; if a raised biomarker level is identified, how should this modify practice to mitigate risk?

*LV = left ventricular*

A *post-hoc* subgroup analysis of the Hip fracture accelerated surgical treatment and care track (HIP-ATTACK) trial ( ClinicalTrials. gov identifier: NCT02027896) found that patients with an elevated baseline troponin had a lower risk of 90 day mortality when surgery was performed within 6 hours of hip fracture diagnosis, compared with the standard of care (hazard ratio [HR] 0.43, 95% confidence interval [CI] 0.24–0.77).^21^ This was in contrast to the overall trial population, where early surgery did not impact 90 day mortality (HR 0.91, 95% CI 0.72 to 1.14).^22^ The authors suggested those with an elevated troponin may represent a high-risk subgroup who could not tolerate the physiological stress from a hip fracture and hence benefit from earlier intervention.^22^

#### Comprehensive pre-operative assessment

Medical optimization should not just be limited to cardiovascular disease; rather, co-morbidities such as diabetes and chronic airways disease should also be optimized to not only reduce long-term cardiac risk, but also potentially reduce surgical stress.^3^ Other factors which may confer risk of a perioperative cardiac event include anaemia and chronic kidney disease.^3^ This is particularly pertinent in older patients (>65 years) who tend to be more frail and multi-morbid; in this setting, a randomized trial (ISRCTN identifier: ISRCTN23142588) on the use of a specialized comprehensive geriatric assessment with medical optimization prior to vascular surgery was found to reduce length of hospital stay and postoperative cardiac complications (25 versus 7%, p<0.001).^23^ The study concludes that this preoperative assessment is best conducted by a dedicated team in a ‘one-stop’ clinic, reducing the need for multiple consultations and parallel care pathways; the study found that two-thirds of patients referred to such a service received a new diagnosis not previously seen, and three-quarters received medication changes as a result of findings from the assessment.^24^

### Surgical risk assessment

**Figure 3:**
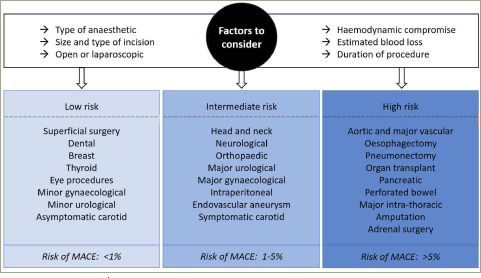
Examples of low/intermediate/high-risk surgeries for estimated risk of perioperative major adverse cardiac events at 30 days

In the UK, of the 2.5 million operations performed annually, 780,000 are classed as moderate-to-high risk, with approximately 12,000 deaths occurring each year as a result of surgery.^25^ Whilst perioperative cardiac complications occur in approximately 5% of patients undergoing elective NCS, this can rise to 40% in high-risk emergency procedures, including major vascular interventions.^26^ Each surgical procedure confers a different degree of stress on the cardiovascular system. Major surgery, in particular intracavity procedures, result in greater fluid shifts, resulting in changes to blood pressure and preload, as well as tissue trauma, which causes a systemic inflammatory response (*Figure 3*).^3^

### Cardiac risk assessment

A range of cardiac investigations are available to assess CAD, including anatomical tests such as computed tomography coronary angiography (CTCA), and stress imaging, including dobutamine stress echocardiography (DSE), single-photon emission computerized tomography (SPECT) and cardiac magnetic resonance imaging (CMR).

#### Anatomical assessment

Following updated National Institute for Health and Care Excellence (NICE) guidelines on the investigation of chest pain of suspected cardiac origin from 2016, the use of CTCA has increased significantly over the past 10 years (Clinical guideline CG95).^27^ A recent review described the risk of perioperative MACE increasing with the degree of CAD on CTCA; 4% when no obstructive disease present, 7% for single vessel disease and up to 23% in multivessel disease.^28^ This suggests that CTCA is useful in ‘ruling out’ relevant CAD in the perioperative setting. Computed tomography (CT) coronary artery calcium (CAC) score also correlated with perioperative risk, and CAC scoring was found to be a successful rule-out test prior to liver transplantation, with the authors concluding patients with a CAC <100 can safely undergo transplantation without further stress testing; however this was a highly pre-selected group and it is notable that participants had a low event rate, with only three MINS events occuring.^29^ Whilst CAC scoring is not discussed in the recent ESC guidelines (2022), it’s predictive or rule-out value in this setting is of particular interest, given the proportion of patients due to undergo NCS who will undergo preoperative CT imaging, and in whom this additional information could be provided without any additional investigations or cost.^30^ A recent study validated this approach, using a simple CAC severity scoring method on non-gated CT chest imaging conducted within 12 months of NCS; they found increasing incidence of 30 day mortality or MI as the score increased.^31^ Addition of the coronary calcium burden score to the RCRI improved the predictive ability for MACE.^30^

(Figure based on data from ESC/ESA guidelines^3^

*ESC = European Society of Cardiology; ESA = European Society of Anaesthesiology; MACE = major adverse cardiac events; NCS = non-cardiac surgery.*

The ESC guidelines recommend CTCA in three scenarios; patients with chronic coronary syndromes with a low or intermediate pre-test probability; as an alternative to ICA in patients with troponin negative chest pain to exclude ACS with low or intermediate pre-test probability of CAD; or lastly in a non-urgent setting for those undergoing intermediate or high-risk surgery and who are unsuitable for functional testing.^3^ One major pitfall with CTCA is the potential for overestimation of disease significance, supporting the principle that anatomical severity does not always correlate with ischaemia. This was described in the coronary CTA VISION trial, however predictive value was improved when combined with functional testing.^32^

#### Quantifying burden of ischaemia

CT-fractional flow reserve (FFR) is a developing technology and offers the potential to assess both anatomical and functional significance with a single non-i nvasive investigation. Krievins at al. undertook a series of studies including patients without cardiac history or symptoms who were due to undergo elective vascular surgery.^33–35^ All patients underwent preoperative CT-FFR , which identified silent ischaemia (CT-FFR<0.80) in 57–68% of patients, and severe ischaemia (CT-FFR<0.75) in 43–53%.^33–35^ No patients had preoperative revascularization, however 33–40% underwent revascularization between 1 and 3 months postoperatively, and when compared with matched controls, these patients had lower MACE rates. Of note, all patients remained free of ACS or chest pain; revascularization decisions were made by a heart team with the recorded indication being silent ischaemia. In the most recent of this series, the 3-year MACE rate was 4% in those who had undergone revascularization following carotid endarterectomy, compared with 17% in controls, with fewer cardiac deaths (HR: 0.20, 95% CI: 0.04–0.95) and MI (HR: 0.16, 95% CI: 0.05–0.54) also observed.^34^

The 2022 ESC guidelines recommend functional testing in patients undergoing high-risk elective surgery who have poor functional capacity, multiple risk factors or previous revascularization, or intermediate risk “when ischaemia is of concern”.^3^ When selecting preferred modality, local availability may dictate choice. In a large meta-analysis comparing all stress modalities with invasive FFR, CMR performed best with the highest specificity on both a per-patient and per-vessel basis.^36^ DSE has also been found to have good predictive value for perioperative MACE.^37^ The ESC guidelines suggest the decision to undertake diagnostic angiography should follow the same principles as in patients with stable angina.^3^ Invasive coronary angiography offers the opportunity for anatomical and functional assessment of CAD, with the added potential to undertake revascularization; however the evidence base for this is limited and conflicted (*Table 2*).^3,10,11^

## Can the risk of cardiac events be modified?

### Coronary intervention

The Coronary artery revascularization prophylaxis (CARP) trial randomized patients with 70% stenosis in at least one coronary artery to revascularization (coronary artery bypass surgery [CABG] or percutaneous coronary intervention [PCI]) or medical therapy, and found no difference in 30 day or long-term MI or mortality after elective major vascular surgery (median follow up 2.8 years) (*Table 2*).^39^ The study population was at low-to-moderate cardiac risk, and therefore had a lower-than-anticipated event rate, making the trial underpowered. The subgroup analysis identified that preoperative angina and exercise ECG ischaemia predicted perioperative MINS, and in turn pre-operative angina and exercise ECG ischaemia, were associated with 20% mortality rate after 1 year, whereas those with inducible anterior wall ischaemia who underwent pre-emptive revascularization had a reduced risk of MINS and all-cause death. However, due to the underpowered nature of CARP, the inference from this sub-group analysis can only be considered hypothesis-generating.^39,40^

**Table 2:** Studies comparing revascularization to medical therapy with 30-day event rates and relative risk^3,10,11^

Study (year)		N	Revasc method	MINS revasc	MINS medical therapy	RR	Mortality revasc	Mortality medical therapy	RR
**Observational studies**									
CASS (1997)^38^		3,368	CABG	0.8%	2.7%	0.3 (95% CI 0.13–0.70)	1.7%	3.3%	0.51 (95% CI 0.26–0.98)
Randomized studies									
CARP (2004)^39^		510	CABG or PCI	11.6%	14.3%	0.81 (95% CI 0.50–1.29)	3.1%	3.4%	0.92 (95% CI 0.34–2.5)
DECREASE-V (2007)^26^		101	CABG or PCI	34.7%	30.8%	1.12 (95% CI 0.64–1.97)	22.5%	11.5%	1.95 (95% CI 0.78– 4.86)

*CABG = coronary artery bypass grafting; CARP = coronary artery revascularization prophylaxis; CASS = Coronary Artery Surgery Study; CI = confidence interval; MINS = myocardial injury after non-cardiac surgery; N = number; PCI = percutaneous coronary intervention; revasc = revascularization; RR = relative risk.*

The DECREASE-V study was a feasibility pilot study in which 101 higher-risk patients (>3 cardiac risk factors) who in addition had extensive ischaemia on stress nuclear imaging or dobutamine stress echocardiography were randomized to either revascularization or medical therapy, prior to high-risk vascular surgery (*Table 2*).^26^ No difference was found in the primary composite endpoint of all-cause death and non-fatal MI at 30 days (43% versus 33%, p=0.30). Whilst the study was underpowered to make any firm conclusions, it reaffirmed the unacceptably high event rate in this cohort, with perioperative cardiac events occurring in over 40% of participants in both groups. It also highlights an important consideration with respect to potential delays in surgery due to revascularization, with two patients suffering ruptured aortic aneurysms in the interval between revascularization and planned surgery, leading to death.^26^

Illuminati et al. randomized 426 asymptomatic patients undergoing carotid endarterectomy to preoperative coronary angiography or no angiography.^41^ Of the 216 patients in the angiography group, 66 underwent PCI, with a subsequent median delay of 4 days to surgery. Patients in the angiography group had a lower rate of MINS compared to the no angiography group (0% versus 4.2%, p=0.01, odds ratio (OR) 0.22, 95% CI 0.06–0.81). It is unclear whether the observed results were due to revascularization or whether awareness of coronary anatomy led to more intensive risk factor management and perioperative care. Moreover, the use of dual anti-platelets in the angiography group may have contributed to reduced ischaemic events.^41^

In summary, in patients with stable CAD undergoing NCS, there is no conclusive evidence that revascularization reduces the risk of perioperative cardiac complications in comparison with optimal medical therapy and risk factor optimization. This finding is consistent with data from landmark trials of revascularization of stable CAD, including the Clinical Outcomes Utilizing Revascularization and Aggressive Drug Evaluation (COURAGE) trial ( ClinicalTrials. gov identifier: NCT00007657),^42^ the International Study of Comparative Health Effectiveness with Medical and Invasive Approaches (ISCHEMIA) trial ( ClinicalTrials. gov identifier: NCT01471522)^43^ and Revascularisation for Ischaemic Ventricular Dysfunction (REVIVED) trial ( ClinicalTrials. gov identifier: NCT01920048)^44^, which have all demonstrated that PCI does not reduce the risk of death or MI compared with optimal medical therapy.^42,43^ The ESC Guidelines make a class IIb recommendation (level of evidence B) to offer preoperative revascularization in the context of significant ischaemic burden, with or without the presence of symptoms, which is based on an individual case-by-case analysis and personalization of preoperative planning.^3^ However, current data mentioned previously does not support routine preoperative revascularization in patients with CAD undergoing NCS in the absence of anginal symptoms.

### Acute coronary syndromes

History of acute MI prior to NCS is associated with increased risk of MINS and death.^45^ Livhits et al. utilized a large database from the USA and found the risk for MINS decreased as time-to-surgery from MI increased, such that within 30 days the risk was 32.8%, falling to 5.9% after 90 days.^46^ In a separate analysis from the same database, the authors found that revascularization for ACS prior to surgery was associated with a reduction in MINS (5.1% versus 10%, p<0.001) and 30-day mortality (5.2% versus 11.3%, p<0.001).^46^ Importantly, if PCI was performed within 30 days of surgery, a trend to recurrent MI was observed (relative risk [RR]: 1.36, 95% CI: 0.96–1.97); in contrast, this risk was significantly reduced with CABG (RR: 0.70, 95% CI 0.55–0.95). Therefore, in the context of an ACS occurring prior to surgery, revascularization should be offered to all patients as per usual practice, and subsequently, surgery delayed for as long as possible.

### Mode of revascularization

PCI is now the most common mode of revascularization used for stable and unstable coronary syndromes, and is associated with shorter recovery times and lower in-hospital major adverse events than bypass surgery.^3^ Although PCI and CABG have not been directly compared in a preoperative setting, retrospective analysis and subgroup analysis of CARP did not demonstrate differences in outcome, aside from a greater delay to NCS in the CABG group (however, as noted previously, this trial was underpowered and clear conclusions cannot be drawn).^40^

Whilst PCI may prevent further delay of the index surgical procedure, it necessitates a period of dual antiplatelet therapy (DAPT), which increases risk of perioperative bleeding complications and may preclude surgery where neuroaxial block is required. Any decisions regarding premature discontinuation of antiplatelet therapy need to be countenanced against the potential harm of acute stent thrombosis. Current guidelines suggest that patients at high bleeding risk can have one antiplatelet agent discontinued as early as 1 month (Class 2b) or preferably 3 months (Class 2a) following elective PCI (*Table 1*) however this decision will also be affected by the complexity of PCI undertaken (left main stem, stent length, bifurcation involvement).^47^ Frequently, the NCS will need to take priority, such as in the setting of urgent cancer surgery. Where it is not possible to delay NCS for more than 30 days, CABG may be the preferred method of revascularization. These discussions should be undertaken by a multi-disciplinary team, where the risk of perioperative complications can be balanced against the potential benefits of revascularization.

## Medical therapy

Despite the above data and guidelines recommendations,^47^ it is notable that over one-third of perioperative MACE events occur in patients with a negative stress test.^28^ Given the uncoupling of demonstrable ischaemia and perioperative events, approaches to reduce risk may need to go beyond revascularization.

Historically there has been controversy regarding perioperative beta blockade. The Dutch Echocardiographic Cardiac Risk Evaluation Applying Stress Echo (DECREASE-I ) trial assessed patients with at least 1 risk factor who were undergoing major vascular surgery, enrolling those who had reversible cardiac ischaemia on DSE.^48^ One hundred and twelve patients were randomized to either standard care, or bisoprolol initiation at least 1 week prior to surgery and for at least 30 days postoperatively, with a lower rate of cardiac death and non-fatal MI seen in the latter (3.4% versus 34%, p<0.001). The study was terminated early due to these marked findings. Whilst these results were subsequently supported by the DECREASE-I V trial (ISRCTN registry identifier: ISRCTN47637497), the results from both trials are nowcalled into question due to suspicion of falsified information.^49,50^

The PeriOperative ISchemic Evaluation trial (POISE) trial ( ClinicalTrials. gov indentifier: NCT00182039) assigned 8,351 patients undergoing NCS to metoprolol or placebo, finding a lower rate of the primary outcome of CV death, MI or cardiac arrest in the intervention arm (HR: 0.84, p=0.0399).^51^ However, there was a higher rate of death and stroke in the metoprolol arm, related to increased rates of severe hypotension and bradycardia. Of note, the trial used a relatively high dose of metoprolol (100 mg), which was given just 2–4 hours before the surgical procedure and which may have impacted the trial outcomes.^51^ Subsequent large metaanalyses have not identified significant differences in mortality or MI outcomes, although they do report a lower incidence of atrial fibrillation or ventricular arrhythmias in patients taking beta blockers.^52,53^

The largest trial in this arena of perioperative stain therapy is the Lowering the Risk of Operative Complications Using Atorvastatin Loading Dose (LOAD) study ( Clinicaltrials. gov identifier: NCT01543555), which randomized statin-naïve, high-risk patients who were scheduled for NCS to atorvastatin or placebo, finding no difference between the groups for a composite outcome of 30-day mortality or MI.^54^ However, a large retrospective analysis of 180,478 patients undergoing NCS found that perioperative statin exposure was associated with a reduction in 30-day mortality (RR: 0.82, 95% CI 0.75–0.89, p<0.01, NNT 67).^55^ The ESC guidelines recommend statin initiation preoperatively if a conventional indication exists.^3^

Glucose management should also be considered perioperatively, as hyperglycaemia may be present, not only in patients with diabetes, but also secondary to a stress response in non-diabetic patients.^56^ A hyperglycaemic state is associated with an increase in the release of inflammatory and vasoconstrictive factors, increased oxidative stress and endothelial dysfunction, and has been linked to increased risk of infection, delayed wound healing and poor cardiovascular outcomes in the setting of acute MI.^56–58^ If identified pre-operatively, glucose levels should be stabilized prior to surgery where possible, however intensive treatment has been linked to poor outcomes in critical care settings, likely related to increased incidence of hypoglycaemia.^59^ Sodiumglucose cotransporter-2 inhibitors have shown excellent results in other settings in recent years, however guidelines current advise that these agents should be held prior to surgery to avoid hypotension.^3,60^ The use of these agents for perioperative optimization may be an area for future research.

## Post-surgical management

A sub-study of the POISE-2 trial identified that any episode of hypotension experienced during the first 4 postoperative days increased risk of MI or death, even when controlling for previous hypotension.^61^ Re-i nitiation of any cardiac medications held during the surgical period will need to be considered. Postoperative ACS should be managed as per the non-surgical setting, although increased bleeding risk and the predominance of type 2 events in the postoperative period should be considered.^47^

Recent guidelines have focused on postoperative troponin monitoring, even in asymptomatic patients (*Table 1*).^3^ If troponin is elevated, clinical assessment, ECG and echocardiography are suggested to help differentiate between type 1 and type 2 MI.^3^ The Vascular events In noncardiac Surgery patIents cOhort evaluatioN (VISION) study ( ClinicalTrials. gov identifier: NCT00512109) assessed 15,065 patients over the age of 45 years, finding 8% had an elevated troponin postoperatively, meeting the criteria for MINS, which was predictive of 30-day mortality.^62^ Whilst identification of higher risk could lead to prolonged care in high-intensity environments, data regarding how best to prevent or manage postoperative troponin rises are sparse.^63^ It is known that elevated serum troponin levels are seen in the setting of severe illness, including sepsis, coronavirus disuease-19 (COVID-19) and acute surgical presentations, which is not always associated with obstructive CAD.^64,65^

A strategy of dabigatran initiation after a perioperative event now also features in guidelines.^3^ This followed publication of the Dabigatran in patients with myocardial injury after non-cardiac surgery (MANAGE) trial ( ClinicalTrials. gov indentifier: NCT01661101), which randomized 1,754 postoperative patients who had experienced MINS to dabigatran or placebo for up to 2years.^66^ The composite primary outcome of vascular mortality, MI, stroke, peripheral thrombosis or amputation was lower in the dabigatran group (11% versus 15%, HR: 0.72, p=0.0115), with no increase in bleeding.^66^

Long term, the occurrence of a perioperative cardiovascular complication, including a detected biomarker rise, could be considered a failed ‘stress test’. Prevention of future events is key, including coronary investigations, linking patients into appropriate outpatient services and specialist follow up. Involvement of a cardiologist in diagnosis and therapy for patients who experienced MINS was found to reduce long-term risk; notably this study was retrospective and therefore the risk of confounding factors and bias is high.^67^

## Conclusions

Cardiovascular complications remain a significant cause of perioperative morbidity and mortality. A combination of patient, surgical and cardiac factors can be used to identify those at highest risk of acute events.

With respect to CAD, there is no strong evidence for preoperative revascularization outside of ARSs. Careful pre-procedural planning, optimization of medical therapy and perioperative monitoring with access to emergency cardiac care are therefore key to reducing cardiovascular events. A randomized trial comparing contemporary revascularization techniques and medical therapy is urgently required to support decisionmaking and treatment in this high-risk cohort.
